# Transcriptomic characterization reveals prognostic molecular signatures of sorafenib resistance in hepatocellular carcinoma

**DOI:** 10.18632/aging.202365

**Published:** 2021-01-20

**Authors:** Wei Yuan, Ran Tao, Da Huang, Weiming Yan, Guanxin Shen, Qin Ning

**Affiliations:** 1 Department and Institute of Infectious Disease, Tongji Hospital, Tongji Medical College, Huazhong University of Science and Technology, Wuhan, China; 2 Department of Immunology, School of Basic Medicine, Tongji Medical College, Huazhong University of Science and Technology, Wuhan, China

**Keywords:** transcriptomics, sorafenib resistance, hepatocellular carcinoma, immune filtrates, bioinformatics

## Abstract

Sorafenib is the first-line treatment for patients with advanced unresectable hepatocellular carcinoma (HCC); however, only a small number of patients benefit from sorafenib, and many develop sorafenib resistance (SR) and severe side effects. To identify biomarkers for SR, we systematically analyzed the molecular alterations in both sorafenib-resistant HCC specimens and cultured cells. By combining bioinformatics tools and experimental validation, four genes (*C2orf27A*, insulin-like growth factor 2 receptor, complement factor B, and paraoxonase 1) were identified as key genes related to SR in HCC and as independent prognostic factors significantly associated with clinical cancer stages and pathological tumor grades of liver cancer. These genes can affect the cytotoxicity of sorafenib to regulate the proliferation and invasion of Huh7 cells *in vitro*. Additionally, immune-cell infiltration according to tumor immune dysfunction and exclusion, a biomarker integrating the mechanisms of dysfunction and exclusion of T cells showed good predictive power for SR, with an AUC of 0.869. These findings suggest that immunotherapy may be a potential strategy for treating sorafenib-resistant HCC. Furthermore, the results enhance the understanding of the underlying molecular mechanisms of SR in HCC and will facilitate the development of precision therapy for patients with liver cancer.

## INTRODUCTION

Hepatocellular carcinoma (HCC) is among the most lethal malignancies, with high morbidity and mortality worldwide. HCC is the second leading cause of cancer-related deaths globally, with only a 5-year survival rate of ~10% and resulting in 700,000 deaths annually [1]. The main risk factors for HCC are hepatitis B or C infection, alcoholism, diabetes, autoimmune hepatitis, and possibly several metabolic diseases. In developed countries, there has been an increase in HCC incidence, partly attributed to obesity and diabetes [2, 3].

Treatment strategies for HCC, including radiofrequency ablation, liver transplantation, or resection, might be curative only when diagnosed early. However, most patients are relatively asymptomatic during early stages, and due to the rapid progression of the disease, 80% of HCC patients are diagnosed at advanced stages. Less than 30% of patients with HCC can be managed by curative therapy, and sorafenib, a multi-target kinase inhibitor, has been approved in a number of randomized controlled trials (RCTs) for first-line treatment in patients with advanced liver cancer to improve overall survival [4, 5]. Sorafenib can block tumor cell proliferation by promoting apoptosis and suppressing anti-apoptotic and metastatic activity; however, the response rate to sorafenib is low, and many patients develop drug resistance and serious adverse side effects. Initiation and development of sorafenib resistance (SR) is considered a multi-step process, although the precise molecular events that underlie resistance remain only partially understood. This highlights the need for continued development of biomarkers allowing determination of which patients are more likely to benefit from sorafenib and a better understanding of the molecular mechanisms involved.

Novel treatments are under intense exploration. Combination with traditional treatment or other drugs has potential clinical utility [6–8]. Histone deacetylase is one of the potential therapeutic targets [9]. Panostatin combined with sorafenib resulted in decreased tumor volume and increased survival in a murine xenograft model [10]. Tegafur/uracil can be used in combination with sorafenib in patients with advanced liver cancer, which can improve the efficacy of sorafenib while showing safety [11]. Recently, immunotherapy based on immune checkpoint inhibitors (ICIs) has delivered unprecedented success. ICIs targeting the programed cell death protein 1/programmed death ligand-1 (PD-L1) pathway demonstrated clinical activity in HCC, whereas many other ICIs are in clinical development [12]. The role of the tumor microenvironment (TME) has received increased attention across a number of cancers in recent years; however, to date, there remains a paucity of discussion concerning cellular characterization of immune infiltrates and relationship between TME and SR in HCC patients treated with sorafenib.

Here, we conducted a comprehensive and integrative analysis, as well as experimental validation, to identify biomarkers predicting SR in HCC and factors independently associated with prognosis. Two microarray datasets from the Gene Expression Omnibus (GEO) were analyzed using a series of bioinformatics tools and another independent dataset from The Cancer Genome Atlas (TCGA) as the validation series. This study provides predictive biomarkers and potential therapeutic targets for SR in HCC patients and might contribute to the development of precision therapy for HCC.

## RESULTS

### Identification of DEGs

**Figure 1:**
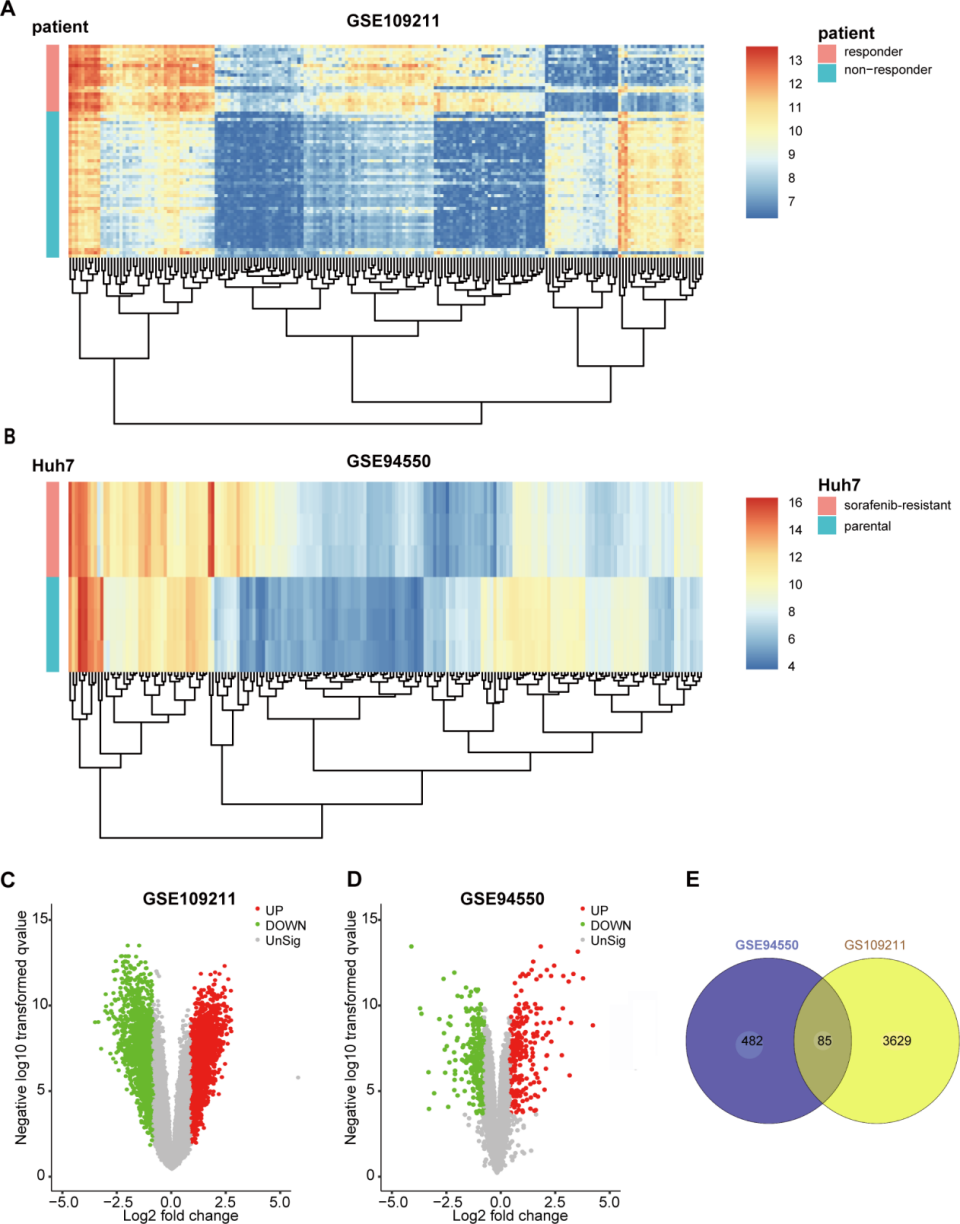
**Identification of DEGs in sorafenib-treated HCC.** To screen the genes associated with resistance or response of sorafenib (|log2FoldChange| ≥1 and FDR <0.05), 3714 DEGs between responder and non-responder were identified following the selection criteria, while 567 DEGs between untreated parental Huh7 cells (n=3) and sorafenib-resistant pool cells (n=3) in GSE94550. The DEGs were visualized with Heatmaps and volcano plots. (**A**) Heatmap plot of the top 200 DEGs of GSE109211. (**B**) Heatmap plot of the top 200 DEGs of GSE94550. (**C**, **D**) Volcano plot of the DEGs of GSE109211 and GSE94550, in which red stands for upregulations, green stands for downregulations, and black stands for normal expression. Each point represents a gene. (**E**) The overlap unified among the 2 datasets contained 85 DEGs was shown by Venn diagram. DEGs, differentially expressed genes.

The GSE109211 dataset contained 140 samples from HCC patients, of whom 67 were treated with sorafenib and 73 with Plac. The sorafenib group was divided into “responder” (*n* = 21) and “non-responder” (*n* = 46) groups in terms of RFS. Compared with the responder group, sorafenib non-responders were defined as patients in whom sorafenib had no effect (those not reaching the median RFS) [13]. To screen genes associated with resistance or response to sorafenib (|log_2_FoldChange| ≥ 1 and false discovery rate < 0.05), 3,714 DEGs between responders and non-responders were identified according to the selection criteria, whereas 567 DEGs were identified between untreated parental Huh7 cells (*n* = 3) and SR pool cells (*n* = 3) in GSE94550. The top 200 DEGs from GSE109211 and GSE94550 are shown in the heatmap (Figure 1A, 1B), with DEGs visualized by volcano plot (Figure 1C, 1D). The overlap of the two datasets included 85 genes (Figure 1E).

### Functional enrichment analysis of the DEGs

**Figure 2:**
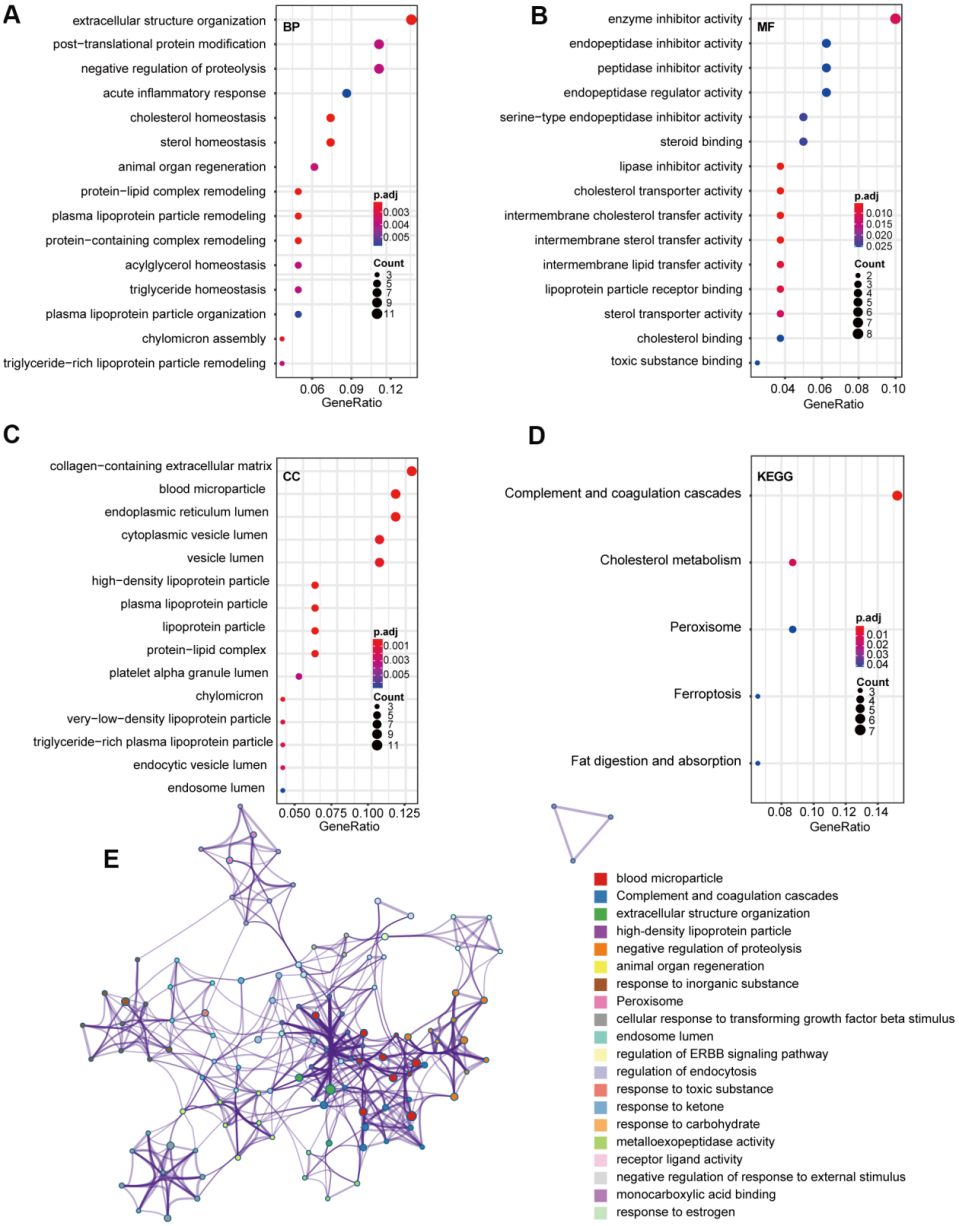
**GO and KEGG pathway analysis of 85 overlap DEGs.** (**A**) Biological process GO terms for DEGs. (**B**) Molecular function GO terms for DEGs. (**C**) Cellular component GO terms for DEGs. (**D**) KEGG analysis for DEGs. (**E**) Enriched Ontology Clusters of statistically enriched terms. Each term is represented by a circle node, where its size is proportional to the number of input genes that fall into that term, and its color represents its cluster identity. The y-axis represents the pathways and the x-axis represents enriched gene numbers, and the color means adjust P-value. The netplot of KEGG pathways means the enrichment of genes in different pathways. And the number adjacent to nodes stands for gene ID. GO, Gene Ontology; BP, biological process; MF, molecular function; CC, cellular component; KEGG, Kyoto Encyclopedia of Genes and Genomes; DEGs, differentially expressed genes.

To explore the potential function(s) of the DEGs, GO and KEGG enrichment analyses were performed. We detected enrichment in several biological process GO terms, such as extracellular structure organization, posttranslational protein modification, and negative regulation of proteolysis (Figure 2A). Regarding molecular function, enzyme inhibitor activity was the most significant (Figure 2B). Furthermore, several cellular components GO terms, such collagen-containing extracellular matrix, blood microparticle, and endoplasmic reticulum lumen, were enriched (Figure 2C). KEGG pathway analysis revealed that complement and coagulation cascades, cholesterol metabolism, and peroxisomes were mostly associated with the DEGs (Figure 2D). To further explore the relationship between these terms, genes were layered into a tree based on Kappa-statistical similarities among their gene members, with terms having a similarity >0.3 connected by edges (Figure 2E).

### Screening of hub genes

**Figure 3:**
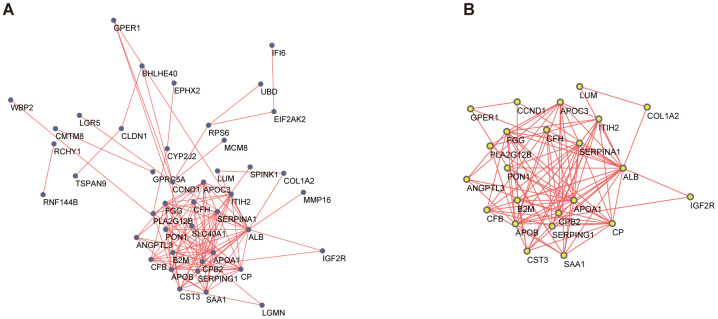
**PPI network and the most significant module of DEGs.** (**A**) The PPI network of DEGs was constructed based on database STRING and Cytoscape software. (**B**) The most significant module was obtained from the PPI network by MCODE with criteria as follows: MCODE scores > 5, degree cut-off = 2, node score cut-off= 0.2, node density cut-off=0.1, Max depth=100 and k-score = 2. PPI, protein-protein interaction; DEGs, differentially expressed genes; MCODE, Molecular Complex Detection (version 1.6), an APP of Cytoscape for clustering a given network based on the topology to find densely connected regions.

A PPI network for the DEGs was constructed by STRING (Figure 3A), with interactions with a combined score >0.4 considered statistically significant. Node pairs were uploaded to Cytoscape and analyzed using MCODE, resulting in 23 hub genes selected from the network according to the following criteria: MCODE score, >5; degree, >2; node score, >0.2; node density, >0.1; and k-score, 2 (Figure 3B).

### CeRNA network

**Figure 4:**
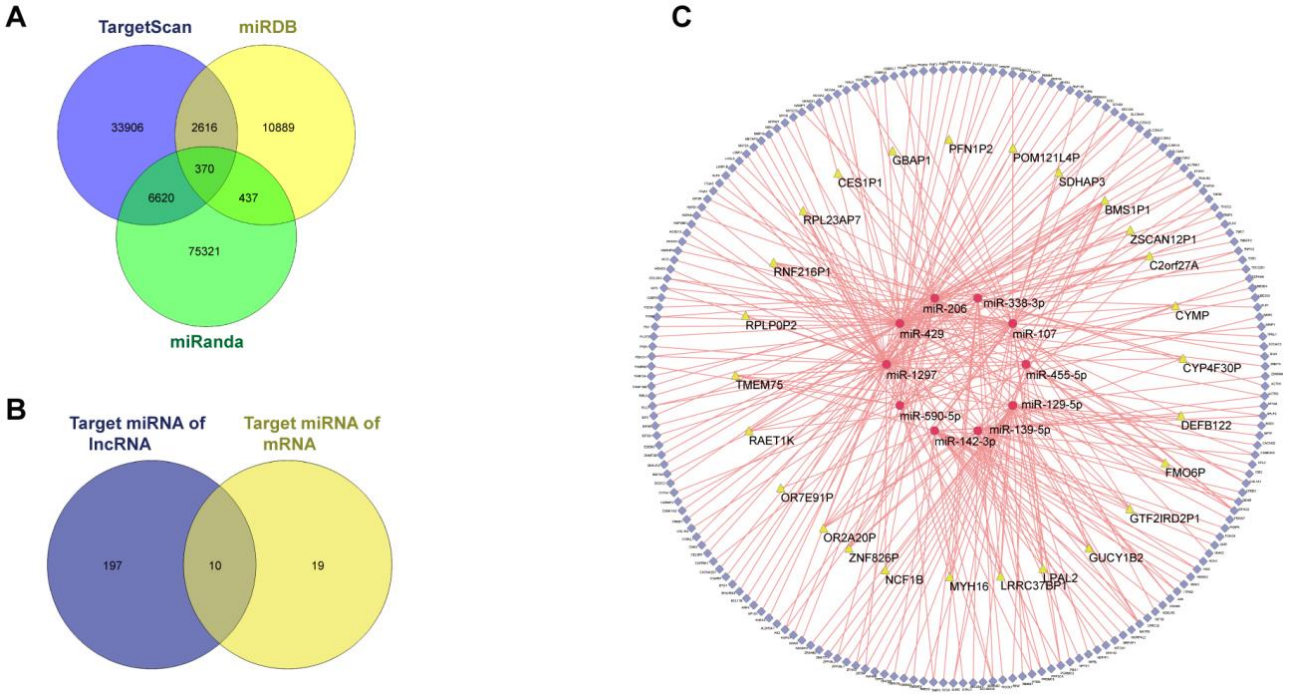
**ceRNA network of GSE109211.** Among the 3714, DEGs between the responder and non-responder in GSE109211, 43 DElncRNAs and 2875 DEmRNAs were identified. miRDB, miRanda, and TargetScan were used to predict the potential miRNA target by DEmRNAs. Only the miRNA-mRNA pairs that exist in all three databases were enrolled in the ceRNA network. The miRcode was applied to predict the potential miRNA target by DElncRNAs. As a result, a total of 26 lncRNAs, 194 mRNAs, and 10 miRNAs were enrolled in the ceRNA network. (**A**) Venn diagram of miRNA-mRNA pairs. (**B**) Venn diagram of target miRNAs. (**C**) CeRNA network of GSE109211. The yellow, red, and blue represent lncRNA, miRNA, and mRNA, respectively. Pink edges indicate lncRNA-miRNA-mRNA interactions. DEGs, differentially expressed genes; LncRNA, long non-coding RNA; miRNA, micro RNA; mRNA, messenger RNA; CeRNA, competing endogenous RNA.

Among the 3,714 DEGs identified between “responder” and “non-responder” groups in GSE109211, 43 DElncRNAs and 2,875 DEmRNAs were identified and annotated. TargetScan, MiRDB, and miRanda were used to predict potential miRNA targets for the DEmRNAs, resulting in formation of 293 of 2,875 DEmRNAs and 29 miRNAs into 370 miRNA–mRNA pairs (Figure 4A). We then used miRcode to identify lncRNA–mRNA pairs, resulting in formation of 27 of 43 DElncRNAs and 207 miRNAs into 1,612 lncRNA–miRNA pairs. We ultimately selected 10 miRNAs (Figure 4B) and finalized the ceRNA network with a total of 26 lncRNAs, 194 mRNAs, and 10 miRNAs (Figure 4C).

### Construction and validation of the lasso regression model

**Figure 5:**
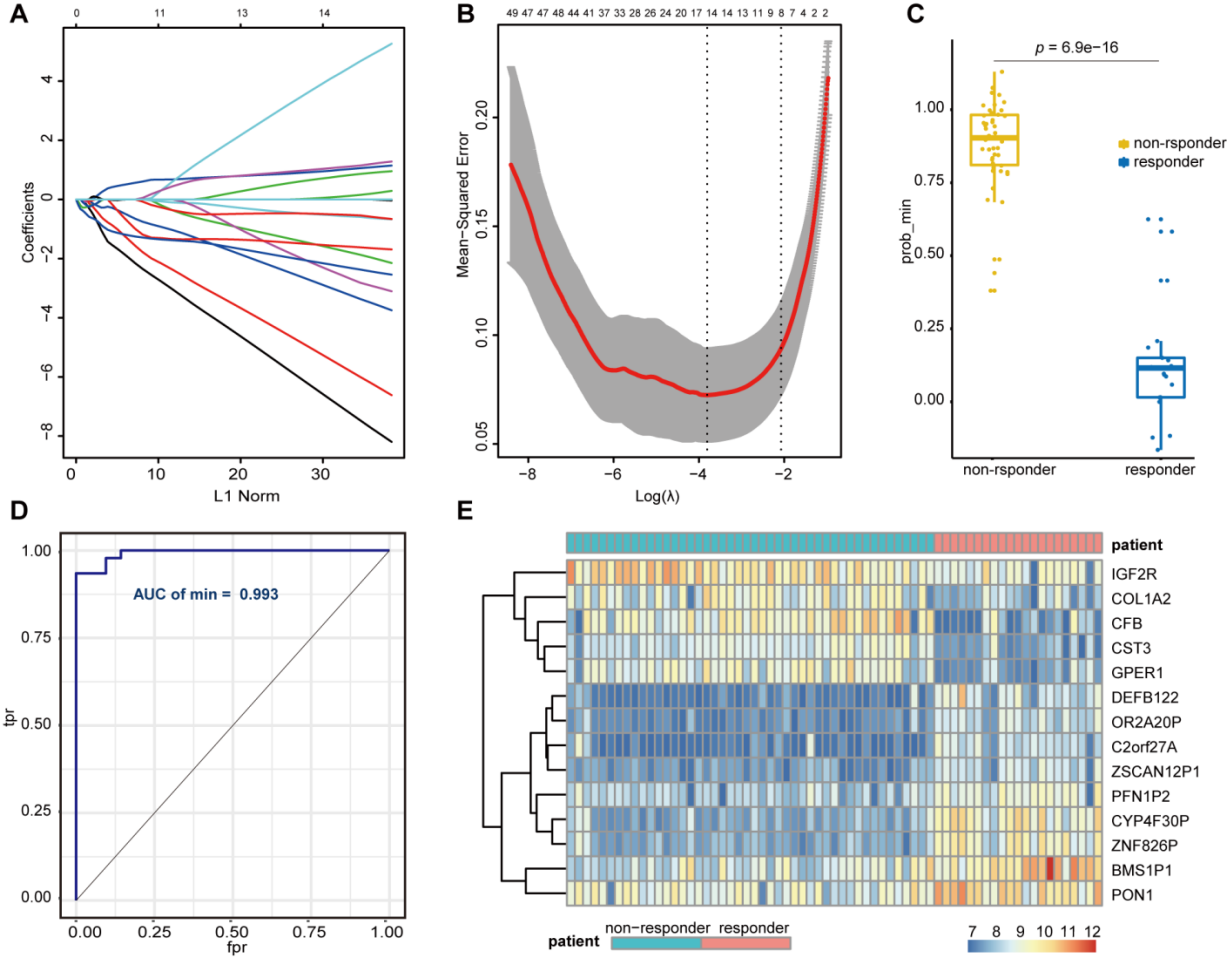
**Lasso regression establishment and validation in GSE109211.** (**A**) LASSO coefficient profiles of the genes associated with the sorafenib resistance in HCC patients. (**B**) The mean-squared error was plotted versus log (lambda). The two dashed lines indicate two special lambda values, one is lambda.min and the other is lambda.1se, and the lambda between the two values is considered appropriate. We finally chose lambda.min, because it’s the value of Lambda that gives a minimum mean cross-validated error. (**C**) Wilcoxon Signed Rank Test was performed to compare the sorafenib non-responder and responder. (**D**) ROC curves for the performance of the Lasso regression model in predicting sorafenib resistance in HCC patients. (**E**) Heatmap of differentially expressed genes that were enrolled in the Lasso regression model. LASSO, Least absolute shrinkage and selection operator; HCC, Hepatocellular Carcinoma; ROC, Receiver Operating Characteristic; tpr, true-positive rates; fpr, false-positive rates.

We divided 67 sorafenib-treated samples from GSE109211 into responder (*n* = 21) and non-responder (*n* = 46) sets according to RFS. To further screen genes associated with SR among the hub genes (mRNA) and the key lncRNAs, we constructed a risk score model of SR prediction using Lasso regression, with the penalty regularization parameter λ determined along with an n-fold value equal to 10 (Figure 5A, 5B). Fourteen genes were identified according to the minimum λ value, including seven mRNAs [*complement factor B* (*CFB*), *collagen type I alpha 2 chain*, *cystatin C*, *G protein-coupled estrogen receptor 1*, *insulin-like growth factor 2 receptor* (*IGF2R*), *profilin 1 pseudogene 2*, *paraoxonase 1* (*PON1*)] and seven lncRNAs (*BMS1P1, C2orf27A, CYP4F30P, DEFB122, OR2A20P, ZNF826P, and ZSCAN12P1*). A Wilcoxon signed rank test performed to compare sorafenib non-responders with responders revealed a significant difference in polygenic risk scores (P = 6.9e−16) (Figure 5C), with the robustness and accuracy of this model indicated according to an AUC of 0.993 (Figure 5D). A heatmap showing the gene expression of selected genes from GSE109211 is shown in Figure 5E.

### TCGA validation of the selected genes

To investigate candidate predictive biomarkers, we analyzed the selected genes against TCGA-LIHC data. Univariate Cox regression performed to screen prognostic factors based on Lasso regression (Supplementary Table 1) revealed four genes selected according to their correlation with overall survival (3 mRNAs and 1 lncRNA). To estimate their potential prognostic value and classification effect in HCC patients, their expression and subsequent protein levels were comprehensively evaluated using UALCAN and the Human Protein Atlas.

**Figure 6:**
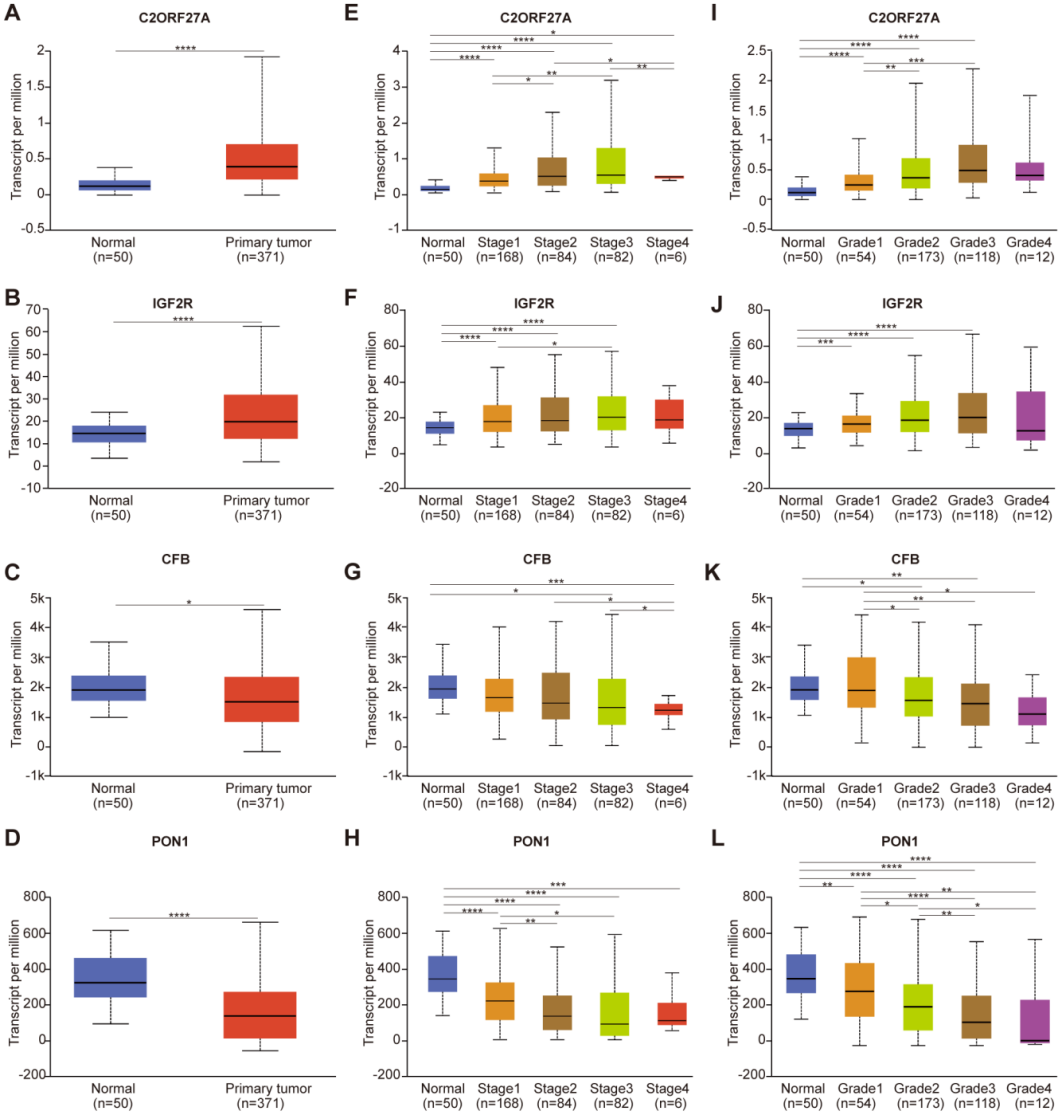
**Relationship between the expression of candidate genes and clinicopathological parameters of HCC patients in TCGA (UALCAN).** (**A**–**D**) Transcriptional expression of candidate genes in HCC tissues and adjacent normal liver tissues. (**E**–**H**) Transcriptional expression of candidate genes in different cancer stages of HCC patients. (**I**–**L**) Transcriptional expression of candidate genes in different tumor grades of HCC patients. HCC, Hepatocellular Carcinoma; *p<0.05, **p<0.01, ***p<0.001, ****p<0.0001.

**Figure 7:**
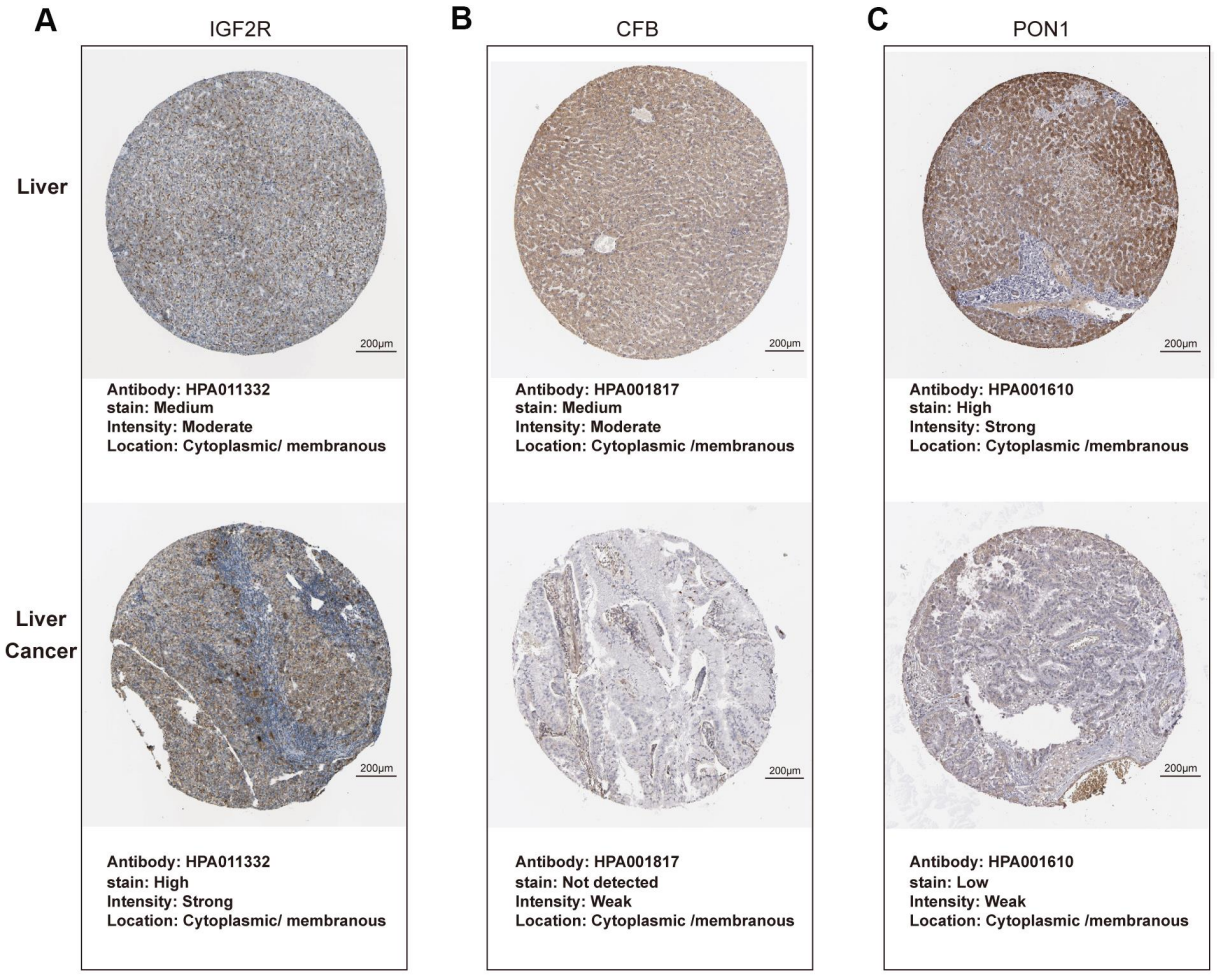
**Protein expression of candidate genes in HCC tissues and normal liver tissues (Human Protein Atlas).** (**A**–**C**) Representative immunohistochemistry images of IGF2R, CFB, PON1 in HCC tissues, and normal liver tissues, respectively.

As shown in Figure 6, expression of *C2orf27A* and *IGF2R* in 20 HCC samples relative to normal tissues was significantly higher according to TCGA datasets (Figure 6A, 6B), whereas *CFB* and *PON1* were significantly downregulated (Figure 6C, 6D). We then assessed protein levels associated with the three mRNAs in HCC cells using the Human Protein Atlas, finding results similar to those for mRNA levels (Figure 7), with elevated IGF2R levels in HCC tissues relative to normal tissues (Figure 7A) and lower CFB and PON1 levels observed in HCC tissues relative to normal tissues (Figure 7B, 7C). These results indicated that *C2orf27A* and *IGF2R* upregulated in HCC patients, whereas *CFB* and *PON1* were significantly downregulated.

We then analyzed relationships between gene expression, cancer stage, and tumor grade. As shown in Figure 6, expression of the four candidate genes was notably correlated with cancer stage and tumor grade, with expression of *C2orf27A* and *IGF2R* higher in patients with advanced cancer or higher tumor grade, whereas *CFB* and *PON1* expression was lower. The highest expression of *C2orf27A* and *IGF2R* was found at stage 3 (Figure 6E, 6F), and the lowest expression of CFB and PON1 was observed at stages 3 and 4 (Figure 6G, 6H), although the reason for higher expression at stage 3 relative to stage 4 might be the small sample size (there were only 6 patients with stage 4 HCC). Similarly, the highest expression of *C2orf27A* and *IGF2R* was found at tumor grade 3 (Figure 6I, 6J), and the lowest expression of *CFB* and *PON1* was found at grade 4 (Figure 6K, 6L). These results identified an association between the expression of four candidate genes with clinicopathological parameters in HCC patients, suggesting their possible involvement in HCC carcinogenesis or progression.

### Prognostic value of the selected genes in liver cancer

**Figure 8:**
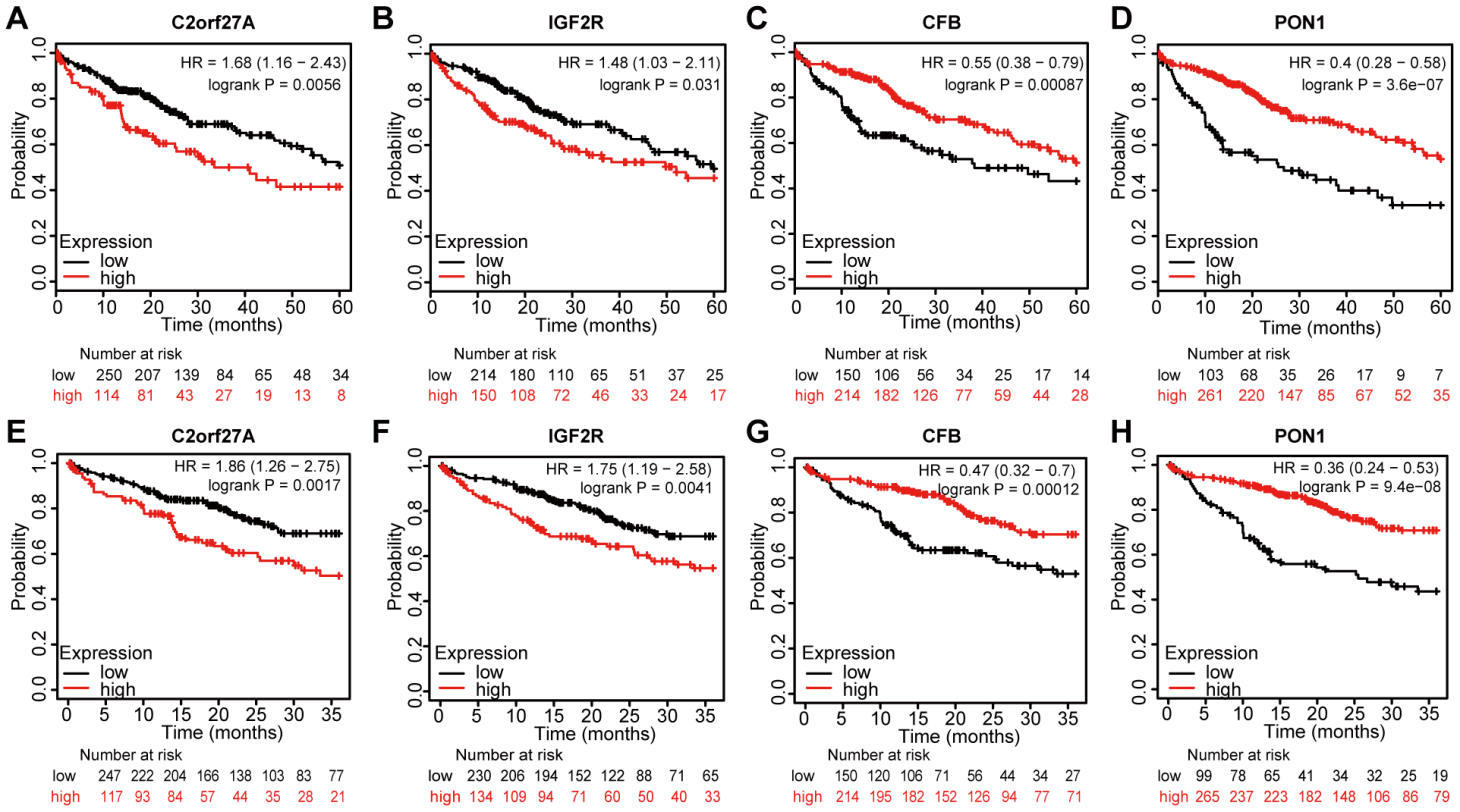
**Prognostic value of selected genes in liver cancer patients (Kaplan-Meier Plotter).** (**A**, **B**, **E**, **F**) Kaplan–Meier survival curves comparing the samples with high expression of the four selected genes with those with low expression in liver cancer conducted within 5 years by using Kaplan–Meier plotter. (**C**, **D**, **G**, **H**) Kaplan–Meier survival curves conducted comparing the samples with high expression of the four selected genes with those with low expression in liver cancer conducted within 3 years by using Kaplan–Meier plotter. HR, hazard ratio.

We assessed the prognostic value of the four genes in liver cancer using Kaplan–Meier analysis. Figure 8 shows the Kaplan–Meier curve and the results of log-rank analyses, revealing that elevated expression of *C2orf27A* and *IGF2R* negatively impacted the 5-year survival rate [hazard ration (HR) =1.68 and 1.48, 95% confidence interval (CI): 1.16–2.43 and 1.03–2.11; p = 0.0056 and p = 0.031, respectively] and 3-year survival rate (HR = 1.86 and 1.75, 95% CI: 1.26–2.75 and 1.19–2.58; and p = 0.0017 and p = 0.041, respectively). Similarly, elevated expression of *CFB* and *PON1* showed positive effects on 5-year survival (HR = 0.55 and 0.4, 95% CI: 0.38–0.79 and 0.28–0.58; p = 0.00087 and p = 3.6e−07, respectively) (Figure 8A, 8B, 8E, 8F) and 3-year survival rates (HR = 0.47 and 0.36, 95% CI: 0.32–0.7 and 0.24–0.53; p = 0.00012 and p = 9.4e−08, respectively) (Figure 8C, 8D, 8G, 8H). These results indicated that the expression of *C2orf27A*, *IGF2R*, *CFB*, and *PON1* were significantly associated with liver cancer prognosis.

### Effect of selected genes on the cytotoxicity of sorafenib

**Figure 9:**
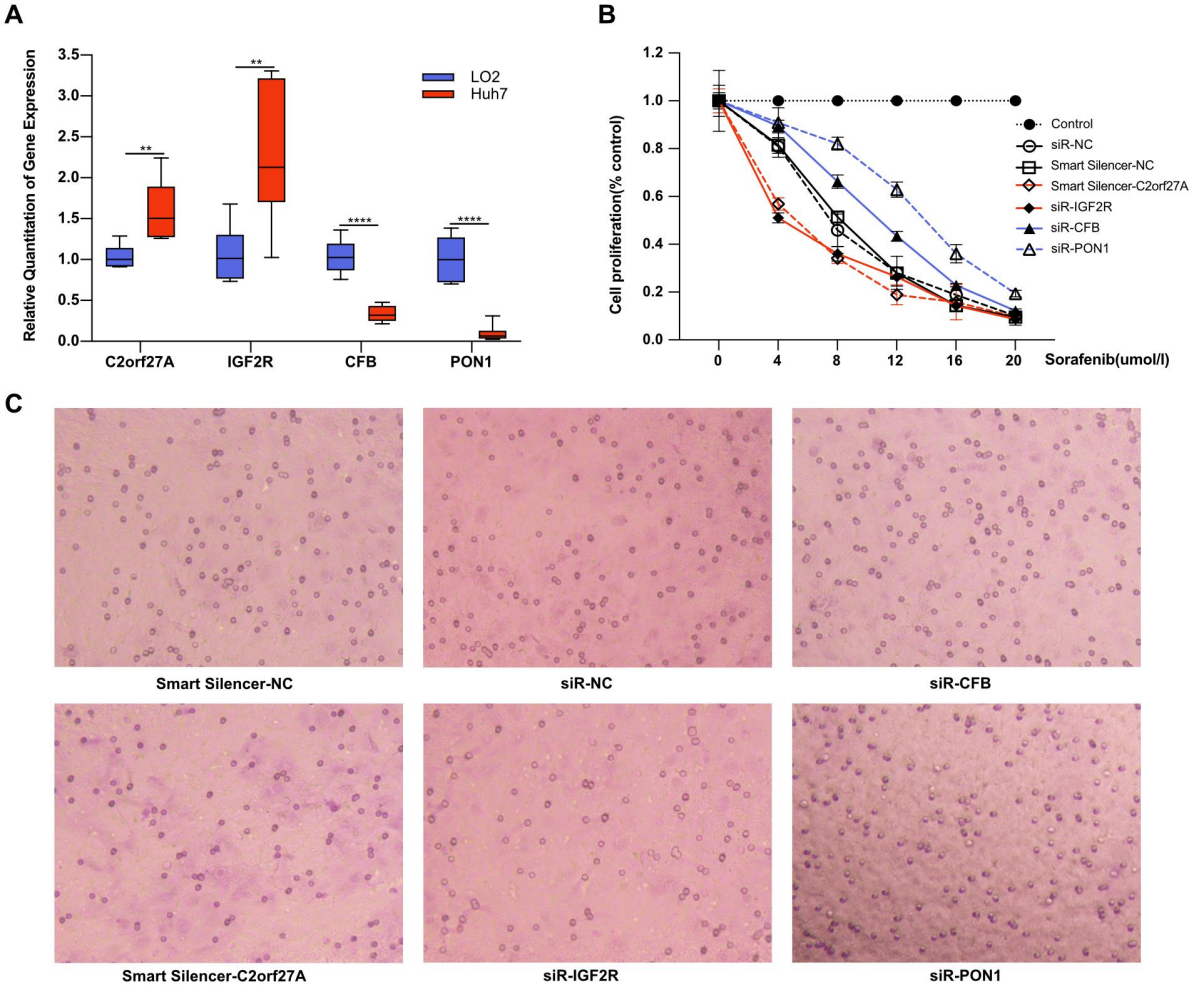
**Effect of selected genes on the cytotoxicity of sorafenib.** Experimental verification of the relationship between the selected genes and sorafenib resistance *in vitro*. (**A**) Transcriptional expression of four selected genes in Huh7 cells compared to LO2. With transfection with siRNA or lncRNA smart silencer, the treated Huh7 cells were exposed to sorafenib, and cell proliferation and invasion were detected 48 hours later. (**B**, **C**) The effects of sorafenib on Huh7 proliferation and invasion after RNA interference. **p<0.01, ****p<0.0001.

To determine whether selected genes were involved in tumorigenesis and development of HCC under sorafenib treatment, we first detected gene expression in liver cell line LO2 and HCC cell line Huh7. As shown in Figure 9A, the expression of *C2orf27A* and *IGF2R* was significantly upregulated in Huh7 cells compared to LO2, and the lower expression of *CFB* and *PON1* was observed in Huh7 cells. We then detected the effects of sorafenib on Huh7 cell proliferation and invasion after RNA interference. Four selected genes were knocked down by transfection with siRNA or lncRNA smart silencer, which were shown in Supplementary Figure 1. The treated Huh7 cells were exposed to sorafenib, and cell proliferation and invasion were detected 48 hours later. As a result, cell proliferation and invasion increased significantly after RNA interference of *CFB* and *PON1*, while significant mitigation was observed after knockdown of *C2orf27A* and *IGF2R* (Figure 9B, 9C), suggesting their possible regulation on the cytotoxicity of sorafenib.

### TME analysis

**Figure 10:**
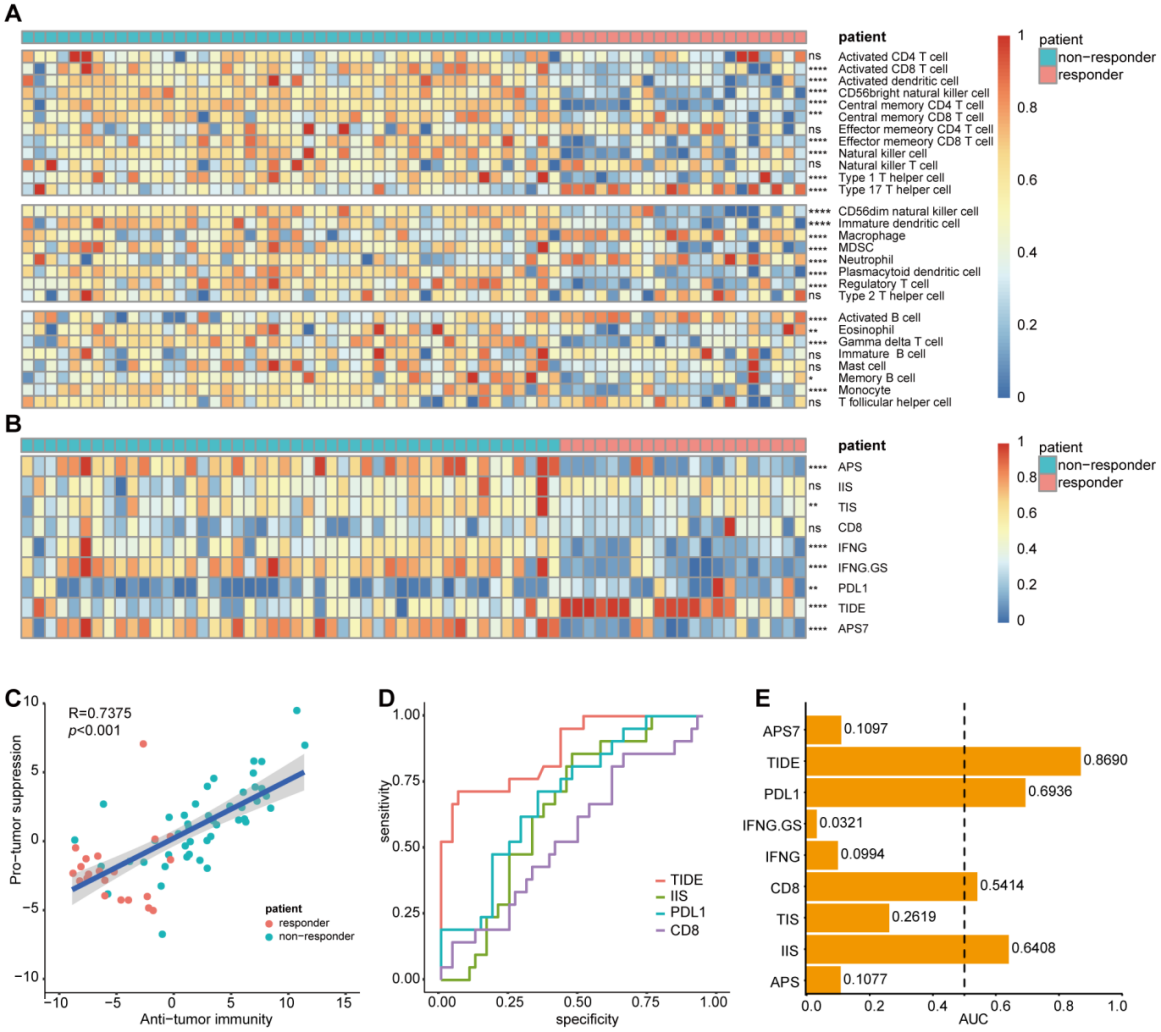
**Correlation of sorafenib resistance and immune cell infiltration heterogeneity.** Single-sample gene set enrichment analysis identifying the relative infiltration of immune cell populations for 67 HCC samples treated with sorafenib in GSE109211. The relative infiltration of each cell type is normalized into a z-score. Biomarkers can predict immunotherapy response were calculated and normalized with GSVA, including APS, IIS, TIS, CD8, IFNG, IFNG.GS, PDL1, TIDE, and APS7. (**A**, **B**) Heatmap of immune cell z-scores and immune infiltration scores. P values describe differences between the responder and non-responder. (**C**) Pearson's correlation between infiltration of cell types executing anti-tumor reactivity (ActCD4, ActCD8, ActDC, TcmCD4, TcmCD8, TemCD4, TemCD8, NKT, NK, CD56^bright^NK, Th1 and Th17 cells) and cell types delivering pro-tumor suppression (CD56^dim^NK, imDC, TAM, MDSC, Neutrophil, pDC, Treg, and Th2 cells). The shaded area represents 95% confidence interval. (**D**) AUC of immune infiltration scores. (**E**) ROC curves for the performance of immune infiltration scores in predicting sorafenib resistance in HCC patients. Only biomarkers with AUC greater than 0.5 were displayed. HCC, Hepatocellular Carcinoma; GSVA, Gene Set Variation Analysis; APS, antigen processing and presenting machinery (APM) score; IIS, immune infiltration score; TIS, T cell infiltration score; TIDE, Tumor Immune Dysfunction, and Exclusion; AUC, Area under the curve; ROC, Receiver Operating Characteristic. ns, no significance, *p<0.05, **p<0.01, ***p<0.001, ****p<0.0001.

We then evaluated the cellular characterization of immune infiltrates in sorafenib-treated patients with HCC. Using an ssGSEA strategy, we estimated 28 immune cell types in the TME, including major types related to adaptive and innate immunity (Figure 10A). To investigate differences in immunophenotypes associated with clinical characteristics of the tumors, variation analysis of the normalized enrichment scores was used to identify SR-associated cell types. As shown in Figure 10A, most tumor-infiltrating lymphocytes (21/28) were associated with SR.

Among the 67 sorafenib-treated patients, the non-responder group displayed increased immune cell infiltration, including antitumor immune cells (ActCD4, ActCD8, ActDC, TcmCD4, TcmCD8, TemCD4, TemCD8, NK cells, NK T cells, CD56^bright^NK cells, and Th1 and Th17 cells) and immunosuppressive cells involved in tumor survival (CD56^dim^NK cells, TAMs, MDSCs, imDCs, neutrophils, pDCs, Tregs, and Th2 cells). Pearson's correlation analysis revealed a significantly positive association between these two types of immune cells within the TME (Figure 10C). These results suggested that antitumor inflammation might promote the differentiation and infiltration of immunosuppressive cells.

Biomarkers predictive of the immunotherapeutic effects of ICIs have been extensively studied and discussed, with their predictive efficacy thought to depend on tumor antigenicity and antigen-presentation efficiency as measured by APS, IIS, TIS, CD8, IFNγ, IFNγ.GS, PDL1, TIDE [14], and APS7 [15]. To explore the potential of immunotherapy in sorafenib-treated patients, these variables were calculated and normalized with GSVA using GSE109211.

IIS results were visualized using a heatmap (Figure 10B), which suggested that most biomarkers predicted a higher potential for ICI immunotherapy in the non-responder group. Additionally, to assess biomarker performance in predicting SR, a ROC curve was used to measure the classification effect at various thresholds. AUCs for these markers were calculated (Figure 10D), with the ROC curves for AUCs >0.5 displayed (Figure 10E). TIDE, a computational framework developed to evaluate the potential of tumor immune escape according to gene-expression profiles in cancer samples, outperformed other biomarkers at predicting SR (AUC = 0.869).

### Correlation between selected genes and infiltrating immune cells

**Figure 11:**
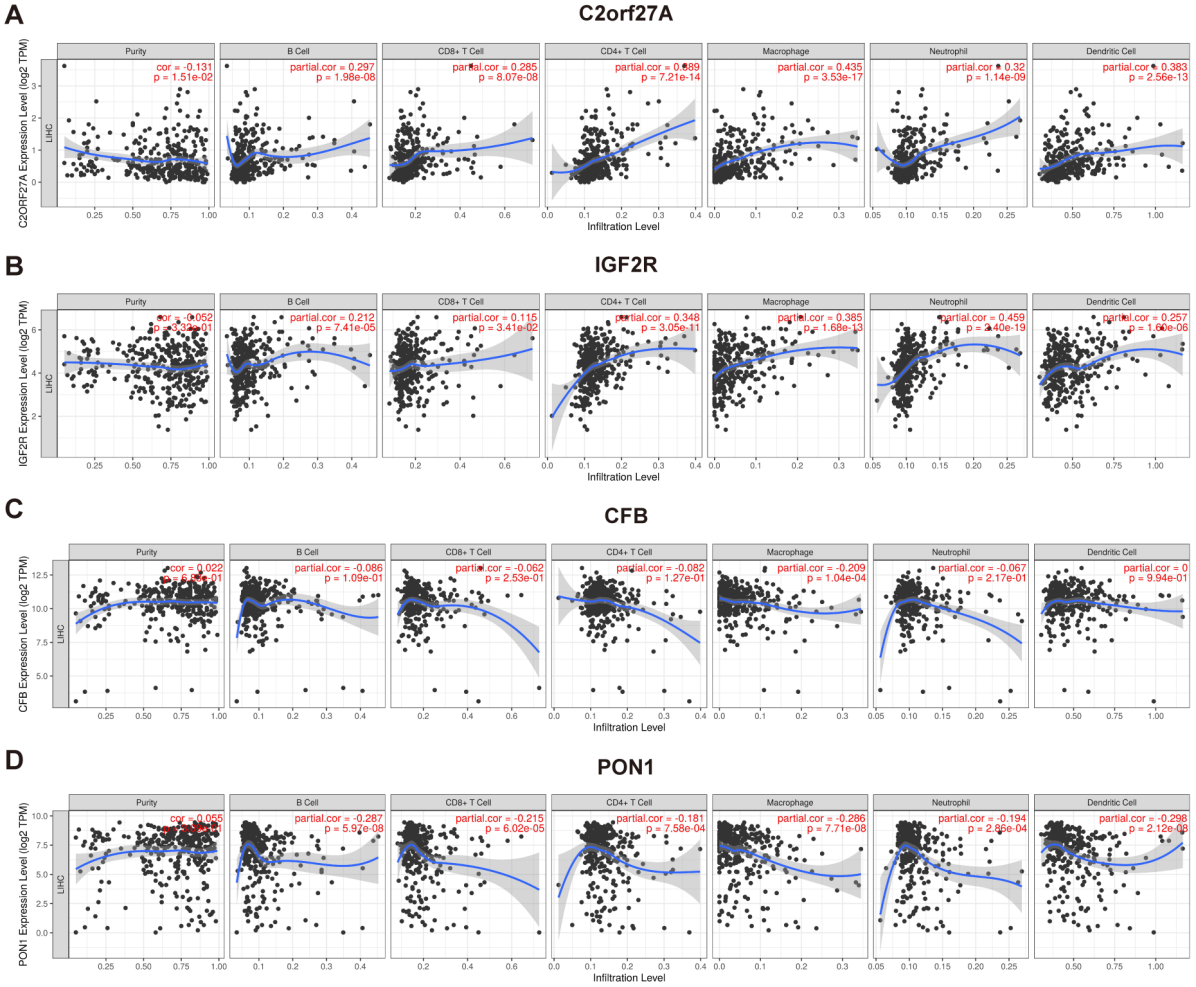
**Association of selected genes' expression with immune infiltration cells.** The correlation between selected genes and immune infiltration in HCC was evaluated by using TIMER (https://cistrome.shinyapps.io/timer/) (**A**) C2orf27a. (**B**) IGF2R. (**C**) CFB. (**D**) PON1. p<0.05 denotes significance. Each dot represents a sample in the TCGA-LIHC dataset.

TIMER was used to investigate correlations between selected genes and immune infiltration in HCC, which includes samples from TCGA (Figure 11). The expression of *CFB* was associated with macrophages, whereas expressions of *C2orf27A*, *IGF2R*, and *PON1* were associated with many immune cells, including neutrophils, macrophages, T cells, B cells, and DCs. These findings indicated that their functions are related to immune regulation in HCC.

## DISCUSSION

In early HCC, surgical resection and local ablation are considered the main treatment modalities, whereas sorafenib is the first-line treatment for patients with advanced unresectable HCC [16]. By inhibiting tumor cell proliferation and angiogenesis, overall survival has been notably lengthened with sorafenib treatment relative to placebo in RCTs [4, 5]. However, it is observed that few patients respond to sorafenib, and its use is associated with severe adverse side effects and drug resistance. Therefore, biomarkers are urgently needed to determine which patients are more likely to benefit from sorafenib. Here, we performed a comprehensive gene expression analysis in both SR HCC patients and *in vitro*.

Due to the genetic heterogeneity of HCC, the acquisition of therapeutic resistance to sorafenib is a serious clinical problem. Recently, microvascular invasion (MVI), high concentration of alpha-fetoprotein (AFP; >200 ng/mL), and a high neutrophil-to-lymphocyte ratio were reported as poor prognostic factors for sorafenib-treated HCC [4, 5, 17]. Additionally, previous studies have shown that overexpression of epidermal growth factor receptor (EGFR) or its ligand in HCC cells might result in continued SR through activation of EGFR downstream signaling. Similarly, these downstream signaling molecules, including Ras/Raf/mitogen-activated protein kinase kinase/extracellular signal-regulated kinase, reportedly affect the sensitivity of HCC to sorafenib [18]. Tasnuva [19] reported a regulatory role of miR-7 in the growth and migration of SR HCC cells by direct inhibition of expression of the tyrosine-protein kinase receptor *TYRO3* and its downstream signaling network. Furthermore, a recent study reported several mechanisms potentially involved in SR, including interaction between the phosphoinositde-3 kinase/Akt and Janus kinase–STAT pathways, the vascular endothelial growth factor (VEGF)-A/VEGF receptor-2 signaling network, autophagy, and the epithelial-to-mesenchymal transition [20–24]. However, their predictive power and molecular mechanisms remain inconsistent and contradictory. In this study, using a combination of liver tissue and cell lines, our analysis eliminated individual patient differences and other factors, such as histories of alcohol and tobacco use, virus infection, family histories, and other treatments, which might have influenced the results. A total of 85 DEGs were screened from the whole genome, and GO analysis revealed that the DEGs were enriched in enzyme inhibitor activity, which is considered to be the main role of sorafenib. Furthermore, sorafenib has recently been reported to downregulate membrane-bound complement regulatory protein to potentiate the antitumor effects of rituximab and ofatumumab in chronic lymphocytic leukaemia [25]. KEGG analysis in this study revealed DEGs are primarily related to complement and coagulation cascades. These findings suggested that the complement pathway may be involved in SR-specific HCC.

We constructed PPI and ceRNA networks to further screen hub genes (mRNA) and lncRNAs. By combining Lasso regression and univariate Cox regression analyses, four key genes related to SR were identified and validated against TCGA data, revealing one lncRNA and three mRNAs exhibiting significantly higher or lower expression in HCC tissues relative to normal tissues. Among the four genes, *C2orf27A* and *IGF2R* were found to be positively correlated and *CFB* and *PON1* were negatively correlated with the stage and tumor grade of liver cancer. Kaplan–Meier analysis subsequently showed that *C2orf27A* and *IGF2R* were negatively correlated with the survival time of liver cancer patients, whereas *CFB* and *PON1* were associated with favorable survival times. Moreover, the role of these genes in SR was also verified *in vitro*. Knockdown of *PON1* and *CFB* significantly attenuated the cytotoxicity of sorafenib in Huh7 cells, while RNA interference of *IGF2R* and *C2orf27A* showed the opposite effect.

Among the four genes, *C2orf27A* has not been previously reported in HCC, whereas *IGF2R* is reportedly related to HCC progression, particularly in regard to transarterial chemoembolisation (TACE) treatment prior to surgery, with TACE-pretreated HCC patients showing significantly higher *IGF2R* mRNA expression in tumor tissues [26]. Additionally, the inactivation of *M6P/IGF2R* occurred in the early stage of hepatocarcinogenesis, which supports the role of *M6P / IGF2R* as a tumor suppressor gene [27]. *CFB* is associated with HBV related HCC [28] and is considered as a potential predictor of response to PegIFNα therapy in patients with chronic hepatitis B [29]. *PON1* is considered as a biomarker for the clinical diagnosis of early HCC and able to distinguish early HCC from liver cirrhosis patients with low AFP levels [30]. Furthermore, *PON1* is potentially related to the MVI of liver cancer [31, 32]; therefore, given the correlation between MVI and sorafenib efficacy [33], *PON1* might play a crucial role in the development of SR by influencing MVI, which agrees with the findings of the present study.

We then investigated the relationship between SR and the TME. Immune cells in the TME can exert both pro- and antitumor effects, with previous studies describing complex interactions between cancer cells and the TME that are implicated in HCC progression [34]. Tumor-associated neutrophils reportedly recruit macrophages and Tregs to promote the growth and development of HCC cells and their resistance to sorafenib [35]. In the present study, we found significant differences in the expression and composition of immune cells between responder and non-responder groups in sorafenib-treated HCC patients. Moreover, compared with the responder group, both antitumor cells and immune cells involved in tumor survival showed higher expression levels in the non-responder group, suggesting the potential efficacy of immunotherapy. Similar results were found for the predictive efficacy of biomarkers for ICI response, reinforcing the potential effectiveness of immunotherapy for treating sorafenib-resistant HCC. Interestingly, the expression of *PD-L1* was lower in non-responders, which might contribute to poor efficacy of *PD-L1* inhibitors. However, without validation, caution must be applied, as these findings might be attributed to the complexity of resistance to cancer immunotherapy. Combination of atezolizumab and bevacizumab has resulted in better overall survival and progression free survival than sorafenib in unresectable HCC [36], which highlighted the immunotherapy based on *PD-L1*. Furthermore, some predictive biomarkers of ICI response showed good predictive power for SR, especially TIDE, a biomarker developed to evaluate the potential of tumor immune escape (ROC = 0.869). This suggests that immune escape might play a crucial role in SR.

Evaluation of the role of selected genes in regulating the immune system with TIMER revealed their association with infiltrating immune cells in HCC tissue samples but not with tumor purity. The results suggest that *CFB* is mainly expressed in macrophages, whereas *C2orf27A, IGF2R*, and *PON1* are widely expressed in many immune cells, including neutrophils, macrophages, T cells, B cells, and DCs. However, only the partial correlation coefficients between *C2orf27A* and macrophages and between *IGF2R* and neutrophils were greater than 0.4. Besides, the correlation analysis between the expression of selected genes and immune cells based on GSE109211 also revealed a close relationship between these genes and the immune system (Supplementary Table 2). These findings indicate that these genes may partly participate in the regulation of immune system in the context of HCC.

This study has some limitations. First, although expression of the four identified genes were designated as predictive biomarkers for SR and independent prognostic factors in HCC patients, the data used in this study were from online public databases based on a retrospective design [13]. Further prospective research with larger sample sizes is needed to validate these findings and explore possible clinical applications of these genes as a therapeutic strategy for HCC. Second, the information from GEO and TCGA might be biased. Although the data were validated in cell lines and clinical specimens, future investigations should expand *in vivo* validation. Finally, potential diagnostic and therapeutic effects have not been assessed for these genes, and additional research is needed to explore whether they are applicable as diagnostic markers or therapeutic targets.

In summary, by combining limma, STRING, MCODE, Lasso regression, and other bioinformatics tools, we identified and characterized several DEGs potentially involved in SR HCC. We identified four genes (*C2orf27A*, *IGF2R*, *CFB*, and *PON1*) as potential predictive biomarkers for SR and independent prognostic factors in HCC. Moreover, we revealed correlations between the genes and clinical cancer stage and pathological tumor grade of liver cancer, and their regulatory effects on sorafenib cytotoxicity were verified *in vitro*. Furthermore, TIDE showed good predictive power for SR, and these results suggest that immunotherapy based on ICIs represents an exciting prospect for treating sorafenib-resistant HCC. These findings may facilitate the development of precision therapy for patients with liver cancer, although future effectors need to be elucidated to fully reveal their contribution to SR in HCC, and validate their usefulness as diagnostic markers or therapeutic targets.

## MATERIALS AND METHODS

### Gene-expression datasets

Gene-expression datasets for HCC patients and a Huh7 cell line were obtained from the GEO repository (http://www.ncbi.nlm.nih.gov/geo), which is an online repository of high-throughput functional genomic data submitted by the scientific community. GSE109211 [13] and GSE94550 were included in this study (GPL13938, Illumina HumanHT-12 WG-DASL V4.0 expression BeadChip; and GPL17586, Affymetrix Human Transcriptome Array 2.0 [HTA-2_0]). The GSE109211 dataset contained liver cancer tissues from 140 patients with liver cancer in the STORM clinical trial (NCT00692770) from 2008 to 2010 and included 67 patients treated with sorafenib and 73 receiving a placebo (Plac). GSE94550 contained Huh7 HCC cells, including untreated parental cells (*n* = 3), SR pool cells (*n* = 3), and SR clone A7 cells (*n* = 3). A7 is a specific clone derived from the SR pool and an *in vitro* model of HCC SR using the Huh7 cell line. Additionally, HCC RNA sequencing (RNA-seq) and clinical data from TCGA data were obtained from the University of California Santa Cruz Xena browser (https://xenabrowser.net/datapages/) [37]. All transcriptome data are freely available online, and this study was performed in compliance with GEO and TCGA data access policies.

### Cell culture and agents

The human HCC cell lines LO2 and HuH-7 were purchased from Cell Bank of Shanghai Institutes for Biological Sciences, Chinese Academy of Sciences (Shanghai, China) and cultured in Dulbecco’s modified Eagle’s medium (DMEM) supplemented with 10% fetal bovine serum (FBS) in a humidified atmosphere of 95% air and 5% CO2 at 37° C. Sorafenib was obtained from MedChemExpress (New Jersey, USA). Cell Counting Kit-8 (CCK8) was purchased from Boster Biological Technology co.ltd (CA, USA). Transwell cell culture plate and matrigel matrix were from Corning (NY, USA).

### Identification of differentially expressed genes (DEGs)

We downloaded the SOFT-formatted family file(s) of datasets from GEO and applied log_2_ conversion to normalize the gene-expression data. DEGs were screened in GSE109211 and GSE94550 using the R package “limma” [38], which is an R/Bioconductor software package used to analyze gene-expression data and especially linear models for microarray data. According to the GTF file (from gencode) and the annotation file, probes IDs were converted to the corresponding sIDs, and genes meeting the cut-off criteria (adjusted P < 0.05; |log_2_| fold change ≥ 1) were considered DEGs. Intersecting portions of the two datasets were detected by Venny 2.1 (https://bioinfogp.cnb.csic.es/tools/venny/index.html).

### Enrichment analysis of DEGs

Cluster Profiler [39] is an R package that implements methods for statistical analysis and allows visualization of biological implications of gene clusters. Metascape [40] is a web-based portal designed to provide free gene annotation and meta-analysis tools (https://metascape.org/gp/index.html). Gene Ontology (GO) enrichment [41] enables gene annotation and analysis of their respective biological roles. The Kyoto Encyclopedia of Genes and Genomes (KEGG) [42] is a database resource that promotes an understanding of high-level functions and biological systems from large-scale molecular datasets generated by high-throughput experimental technologies. To elucidate the biological function of DEGs, “clusterprofiler” and Metascape were used to perform GO and KEGG enrichment analyses.

### Protein–protein interaction (PPI) network construction and DEG module analysis

Search Tool for the Retrieval of Interacting Genes (STRING; http://string-db.org; v.11.0) [43] is a biological database and web resource of known and predicted PPIs. Cytoscape (v.3.7.2) [44] is a bioinformatics software platform used to visualize complex networks. MCODE (v.1.6) [13] is a Cytoscape application used to cluster a given network in order to identify core modules with dense connections. In this study, STRING and Cytoscape were combined to construct and visualize a PPI network, and clustered subnetworks of highly interconnected nodes from the PPI network were identified using MCODE. The screening criteria were as follows: MCODE score, >5; degree cut-off, 2; and node score cut-off, 0.2.

### Integrative analysis of the competing endogenous (ce)RNA network

GPL13938 (GSE109211 platform) was designed to obtain whole-genome expression profiling of samples. Differentially expressed long noncoding RNAs (DElncRNAs) were identified and annotated using the annotation file in GTF format (gencode.v33.annotation.gtf) from the DEGs of GSE109211. Relevant lncRNA-targeted microRNAs (miRNAs) were obtained from the miRcode database (http://www.mircode.org/) [45]. Furthermore, putative target miRNAs of the DEmRNAs were predicted using three databases: miRDB (http://www.mirdb.org/) [46], miRanda (http://www.microrna.org/) [47], and TargetScan (http://www.targetscan.org) [48]. To improve analytical reliability, only target miRNAs existing in all four databases were included in the lncRNA–miRNA–mRNA network. The ceRNA network was visualized using Cytoscape.

### Establishment and validation of lasso regression

HCC samples trained with sorafenib in GSE109211 were divided into responder and non-responder sets according to relapse-free survival (RFS). A risk score model of SR prediction was constructed using the Lasso method with the R package “glmnet” [49], and the penalty regularization parameter lambda (λ) was determined along with an n-fold value equal to 10. The minimal λ value was used to identify key genes. We then used the R package “pROC” [50] to evaluate the robustness of the risk score model in terms of area under the receiver operating characteristic (ROC) curve (AUC).

### Biomarker screening using cox risk regression analysis

According to the RNA-seq and clinical data from TCGA-Liver Hepatocellular Carcinoma (LIHC), univariate Cox regression was performed using the "survival" R package for further variable selection to improve the predictive accuracy and interpretability of biomarkers.

### UALCAN

UALCAN (http://ualcan.path.uab.edu) [51] is an interactive data-mining platform used to analyze cancer transcriptomes in TCGA. We applied UALCAN to analyze the relative expression of candidate genes between HCC and normal samples, as well as associations between gene expression and related clinicopathological parameters, in TCGA.

### The human protein atlas

The Human Protein Atlas (https://www.proteinatlas.org/) is an open-access interactive database used to explore detailed information concerning protein expression and localization according to semiquantitative immunohistochemical analyses of 17 different cancer types [52]. In this study, immunohistochemistry images of protein levels between normal liver tissues and HCC tissues were compared using the Tissue Atlas (https://www.proteinatlas.org/humanproteome/tissue) and Pathology Atlas (https://www.proteinatlas.org/humanproteome/pathology).

### Kaplan–Meier analysis

The Kaplan–Meier plotter (http://kmplot.com/analysis/) allows visualization of Kaplan–Meier survival curves based on data from GEO, the European Genome-Phenome Archive, and TCGA [53, 54]. Tumor patients were divided into high-expression and low-expression groups based on median values of mRNA expression, and the prognostic value of the selected genes in liver cancer was evaluated.

### Real-time PCR

Total RNA of cultured cells or liver tissue was extracted using TRIzol (Invitrogen, NY, USA) according to the manufacturer's instruction. Duplicate samples were subjected to a quantitative real-time polymerase chain reaction (QRT-PCR). Then 1μg of total RNA in a 20μl reaction volume was reverse transcribed into cDNA using ReverTra Ace® qPCR RT Kit (TOYOBO, Japan), and subjected to quantitative PCR using SYBR Green Realtime PCR Master Mix (TOYOBO, Japan) with β-actin as an internal control. All primers are shown in Supplementary Table 3. Thermal cycling consisted of 55° C for 30 min, 95° C for 15 min, 40 cycles at 94° C for 30 s, 60° C for 30 s, and 72° C for 1 min.

### RNA interference

To determine the role of selected genes in sorafenib treatment, Huh7 cells were transfected with siRNA or lncRNA smart silencer according to the manufacturer’s protocol. Briefly, cells were grown to 60 to 70% confluence and incubated with siRNAs or lncRNA smart silencer at a final concentration of 50 nM using LipofectamineTM 3000 (Invitrogen, NY, USA) in serum-free medium for 24 h. SiRNA and lncRNA Smart Silencer were designed and synthesized by RiboBio co.ltd (Guangzhou, China), and the sequences are shown in Supplementary Table 4.

### Cell proliferation and cell invasion assay

The effect of selected genes on cell proliferation was determined by Cell counting kit 8 assay. Cells were seeded at 5000 cells/well and cultured under various concentrations of sorafenib in 96-well plates. After 48h, 10 μl of the CCK-8 solution was added and incubate for 1-4 hours in the incubator, and finally measure the absorbance at 450 nm using a microplate reader.

Cell invasion assays were performed using a Matrigel-coated transwell invasion chamber. Briefly, the cells were pretreated with RNA interference and exposed to sorafenib at 6 μmol/L for 48h. Next, the cells were resuspended in DMEM containing 0.1% bovine serum albumin and added to the upper chambers at a concentration of 0.5×10^5^ cells per well in 24-well plates. DMEM containing 10% FBS was added to the lower chambers as a chemoattractant. After 24 h, the invaded cells on the membrane’s undersurface were stained with 0.1% crystal violet.

### TME analysis

The abundance of 28 immune cells in the TME [24] was quantified by single-sample gene set enrichment analysis (ssGSEA) [55, 56]. Microarray data from GSE109211 were modified and applied using the CIBERSORT with deconvolution algorithm [57]. The ssGSEA score was calculated and normalized between 0 and 1. Additionally, correlations between the infiltration of cell types involved in antitumor immunity and those involved in tumor survival were estimated by Pearson's correlation analysis. The antitumor group included the following cells: activated T cells (ActCD4 and ActCD8), activated dendritic cells (ActDCs), central memory T cells (TcmCD4 and TcmCD8), effector memory T cells (TemCD4 and TemCD8), natural killer (NK) T cells, NK cells (CD56^bright/dim^NK cells), and T helper 1 (Th1) and Th17 cells. The tumor-survival group comprised the following immune cells: CD56dimNK, immature DCs (imDCs), tumor-associated macrophages (TAMs), myeloid-derived suppressor cells (MDSCs), neutrophil, plasmacytoid DCs (pDCs), regulatory T cells (Tregs), and Th2 cells [56].

### Calculation of immune-infiltration score

Biomarkers predictive of the immunotherapeutic effects of ICIs have been extensively studied and discussed, including the antigen processing and presenting machinery scores (APS); T cell-infiltration score (TIS); immune infiltration score (IIS); CD8, interferon γ (IFNγ), the ratio of IFN signaling in immune cells (IFNγ.GS), and PD-L1; tumor immune dysfunction and exclusion (TIDE) [15]. Besides, we conducted another analysis about calculating APS based on 7 genes from Senbabaoglu et al. [58], which were involved in the processing and presentation of antigens on MHC. We named this score as ‘APS7’. APS, APS7, IIS, and TIS were quantified using the R package “GSVA” [59] and gene lists according to a previous report [58]. IIS was defined as the mean of the standardized values of immune cells, including macrophages, DCs, B cells, eosinophils, mast cells, neutrophils, NK cell subsets, and all T cell subsets. TIS was defined as the mean of the standardized values for the following T cell subsets: CD8^+^, T central and effector memory cells, Th1, Th2, Th17, and Treg cells.

The TIDE score [14] was calculated online (http://tide.dfci.harvard.edu) and integrates the expression signatures of T cell dysfunction and exclusion in order to model tumor immune evasion. The calculation of scores for other biomarkers (IFNγ, CD8, PD-L1, and IFNG.GS) has been reported previously [14, 60, 61]. According to the list of genes defined in previous studies, the scores of biomarkers were quantified as the average expression level of the gene set. IFNγ-related biomarkers included C*-X-C chemokine ligand* (*CXCL*)*10*, *CXCL9*, *IFNγ*, *signal transducer and activator of transcription 1* (*STAT1*), *indoleamine 2,3-dioxygenase 1*, and *human leukocyte antigen–DR isotype α*. CD8-related biomarkers including the genes *CD8A* and *CD8B*. Immune scores were normalized to a uniform distribution (range: 0–1), and the AUC for each biomarker was calculated.

### Correlation analysis between selected genes and infiltrating immune cells

To investigate correlations between selected genes and immune infiltration in HCC, we used TIMER (https://cistrome.shinyapps.io/timer/) [62], which includes 10,897 samples across diverse cancer types from TCGA, to evaluate levels of six types of tumor-infiltrating immune cells (B cells, CD4 T cells, CD8 T cells, macrophages, neutrophils, and DCs).

### Statistical analysis

Most of the statistical analyses were conducted using the bioinformatic tools mentioned above. As to experimental validation, data were analyzed and visualized by using GraphPad Prism 8 software. Independent-sample t-test or Mann–Whitney U test was done for independent variables. Differences were considered statistically significant if p <0.05 *; p <0.01 **; p <0.001***; p<0.0001****. Results are expressed as the mean ± standard error of mean.

## Supplementary Material

Supplementary Figure 1

Supplementary Tables
