# Supplementary material for: Transcriptomic characterization reveals prognostic molecular signatures of sorafenib resistance in hepatocellular carcinoma
Source: Aging (Albany NY). 2021 Jan 20;13(3):3969–93. doi: 10.18632/aging.202365 (PMC7906139; doi:10.18632/aging.202365)
Supplement: Supplementary Figure 1 [file aging-13-202365-s001.pdf]

SUPPLEMENTARY FIGURE

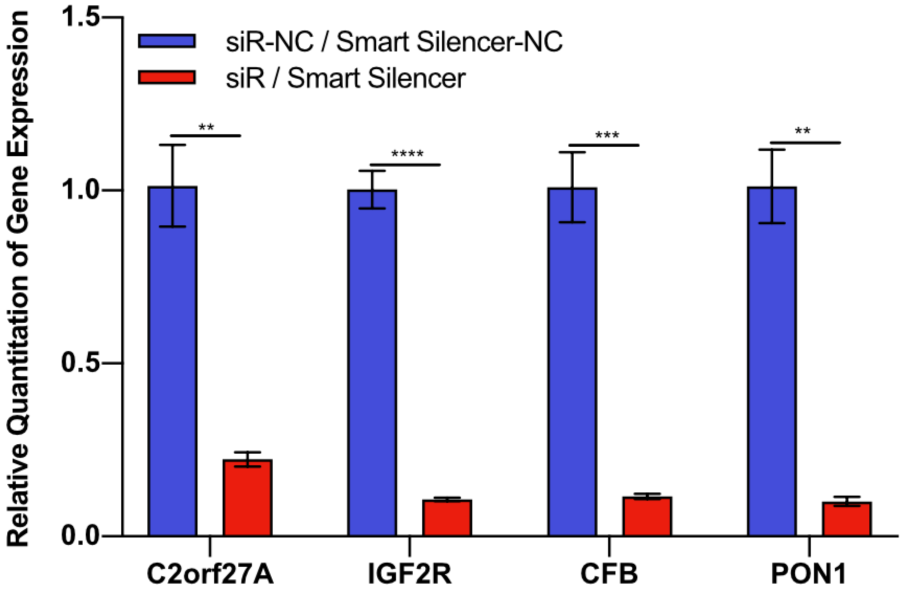

Supplementary Figure 1. Four selected genes were knocked down by transfection with siRNA or lncRNA smart silencer - Four selected genes were knocked down by transfection with siRNA or lncRNA smart silencer. The gene expression of C2orf27A, IGF2R, CFB, and PON1 in Huh7 cells after RNA interference.
