# Supplementary material for: Transcriptomic characterization reveals prognostic molecular signatures of sorafenib resistance in hepatocellular carcinoma
Source: Aging (Albany NY). 2021 Jan 20;13(3):3969–93. doi: 10.18632/aging.202365 (PMC7906139; doi:10.18632/aging.202365)
Supplement: Supplementary Tables [file aging-13-202365-s002.pdf]

SUPPLEMENTARY TABLES

Supplementary Table 1. Cox regression analysis.

	Univariate			Multivariate		
	**Hazard_ratio**	**CI95**	**p_values**	**Hazard_ratio**	**CI95**	**p_values**
Age	1.01	1-1.03	0.066			
Gender	0.82	0.58-1.17	0.284			
Race	1.14	0.95-1.37	0.166			
Family_History	1.16	0.81-1.68	0.418			
BMI	1	0.97-1.03	0.995			
Ethnicity	1.11	0.49-2.52	0.811			
Fibrosis_Stage	1.18	0.87-1.6	0.287			
Child_Pugh_Classification	1.52	0.84-2.77	0.17			
**Tumor_Stage**	**1.64**	**1.34-2.01**	**0**	**0.8**	**0.34-1.87**	**0.611**
**Pathologic_T**	**1.63**	**1.37-1.94**	**0**	**1.95**	**0.87-4.34**	**0.103**
**Pathologic_N**	**1.22**	**1.01-1.46**	**0.037**	**1.07**	**0.81-1.4**	**0.637**
**Pathologic_M**	**1.26**	**1.05-1.52**	**0.012**	**1.24**	**0.95-1.63**	**0.11**
Radiation_Therapy	0.84	0.21-3.42	0.808			
Vascular_Invasion	0.74	0.53-1.02	0.069			
BMS1P1	1.19	0.59-2.4	0.622			
**C2orf27A**	**2.13**	**1.51-2.99**	**0**	**1.5**	**0.99-2.27**	**0.057**
**CFB**	**0.85**	**0.75-0.97**	**0.017**	**1**	**0.85-1.18**	**0.979**
COL1A2	1.06	0.95-1.17	0.291			
CST3	1.2	0.97-1.48	0.091			
CYP4F30P	116840.6	0-4.11e13	0.245			
DEFB122	2384.77	0.03-2.03e8	0.179			
GPER1	0.96	0.81-1.13	0.6			
**IGF2R**	**1.28**	**1.01-1.63**	**0.041**	**1.08**	**0.83-1.4**	**0.565**
OR2A20P	5.13	0.3-86.48	0.256			
PFN1P2	0.33	0.07-1.45	0.141			
**PON1**	**0.85**	**0.79-0.92**	**0**	**0.88**	**0.8-0.97**	**0.01**
ZNF826P	1.35	0.99-1.83	0.057			
ZSCAN12P1	0.94	0.58-1.5	0.784			

Supplementary Table 2. Correlation between selected genes and infiltrating immune cells in GSE109211.

	CFB		IGF2R		PON1		C2orf27A	
	r	p	r	p	r	p	r	p
**Activated.CD4.T.cell**	-0.29	0.02	0.10	0.40	-0.16	0.19	0.19	0.12
**Activated.CD8.T.cell**	0.51	0.00	0.33	0.01	0.09	0.45	0.40	0.00
**Activated.dendritic.cell**	0.43	0.00	0.34	0.00	0.20	0.11	0.09	0.46
**CD56bright.natural.killer.cell**	0.68	0.00	0.56	0.00	0.33	0.01	0.35	0.00
**Central.memory.CD4.T.cell**	0.65	0.00	0.59	0.00	0.41	0.00	0.48	0.00
**Central.memory.CD8.T.cell**	0.44	0.00	0.50	0.00	0.48	0.00	0.09	0.46
**Effector.memeory.CD4.T.cell**	-0.18	0.13	-0.22	0.07	-0.48	0.00	0.02	0.89
**Effector.memeory.CD8.T.cell**	0.70	0.00	0.16	0.20	0.02	0.86	0.35	0.00
**Natural.killer.cell**	0.70	0.00	0.14	0.27	0.04	0.75	0.35	0.00
**Natural.killer.T.cell**	-0.40	0.00	-0.11	0.36	-0.01	0.96	-0.23	0.06
**Type.1.T.helper.cell**	0.56	0.00	0.26	0.03	0.21	0.08	0.44	0.00
**Type.17.T.helper.cell**	-0.52	0.00	-0.33	0.01	-0.30	0.01	-0.44	0.00
**CD56dim.natural.killer.cell**	0.61	0.00	0.13	0.28	0.14	0.26	0.23	0.06
**Immature.dendritic.cell**	0.24	0.05	0.50	0.00	0.48	0.00	0.30	0.01
**Macrophage**	-0.35	0.00	-0.30	0.01	-0.09	0.46	-0.06	0.62
**MDSC**	0.53	0.00	0.56	0.00	0.30	0.01	0.28	0.02
**Neutrophil**	-0.50	0.00	-0.33	0.01	-0.16	0.19	-0.21	0.09
**Plasmacytoid.dendritic.cell**	0.68	0.00	0.66	0.00	0.47	0.00	0.32	0.01
**Regulatory.T.cell**	0.56	0.00	0.13	0.31	0.01	0.95	0.38	0.00
**Type.2.T.helper.cell**	-0.46	0.00	-0.17	0.17	-0.15	0.22	0.02	0.86
**Activated.B.cell**	-0.42	0.00	-0.67	0.00	-0.60	0.00	-0.10	0.41
**Eosinophil**	0.16	0.18	0.34	0.00	0.34	0.01	0.12	0.33
**Gamma.delta.T.cell**	0.65	0.00	0.57	0.00	0.46	0.00	0.38	0.00
**Immature..B.cell**	-0.27	0.03	-0.27	0.03	-0.38	0.00	-0.04	0.74
**Mast.cell**	-0.16	0.20	0.02	0.86	0.02	0.85	0.00	0.99
**Memory.B.cell**	0.24	0.05	-0.00	0.97	-0.10	0.41	0.19	0.13
**Monocyte**	0.75	0.00	0.57	0.00	0.43	0.00	0.38	0.00
**T.follicular.helper.cell**	-0.22	0.08	0.32	0.01	0.16	0.19	-0.21	0.09

Supplementary Table 3. qRT-PCR primers.

Genes	Primers	Sequences (5' to 3')
Homo *β-actin*	Forward	CACCAACTGGGACGACAT
	Reverse	ACAGCCTGGATAGCAACG
Homo *C2orf27a*	Forward	TCACGCTGCTGTGGAATAGAA
	Reverse	ATCAGAGGCTGCTGGGGAA
Homo *IGF2R*	Forward	CACCAGGCGTTTGATGTTGG
	Reverse	TTTGGGAATGGTGCCCTCTC
Homo *CFB*	Forward	TCTCTGTGGCATGGTTTGGG
	Reverse	CCATACAGCTCTCGTGTCCC
Homo PON1	Forward	AGCGTGGTCGTATGTTGTCT
	Reverse	TGAGCCAGCAACTCAGCTAT

Supplementary Table 4. Target sequences of siRNAs and lncRNA smart silencer.

siRNAs	Target sequences
siR-*IGF2R*	GCATCAAGATATCGACTCT
siR-*CFB*	GTGGCAAGTTATGGTGTGA
siR-*PON1*	GTCGTATGTTGTCTACTAT
SmartSiliencer-*C2orf27A* (mix)	
ASO-h-C2orf27A_001	CCAAAGGTTTATGGCTGCAA
ASO-h-C2orf27A_001	GAGAGTAACTTTGTACCCAT
ASO-h-C2orf27A_001	ACCACAGTTACTTTATCCCT
si-h-C2orf27A_001	TCATCTGCGTTGCTCTAAA
si-h-C2orf27A_002	CCTCCAGGTTCAACAGTAT
si-h-C2orf27A_003	ACTGATTCCTTCCGAGACT
